# Bioactive Compounds from Medicinal and Edible Plants for Anti-Aging: A Systematic Review of Molecular Mechanisms, Delivery Systems, and Clinical Evidence

**DOI:** 10.3390/foods15152646

**Published:** 2026-07-28

**Authors:** Zhenyu Ma, Bixue Huang, Kunzhui Chen, Runtian Yuan, Linning Li, Yingbin Shen

**Affiliations:** 1 School of Life Sciences, Guangzhou University, Guangzhou 510006, China; 18219167618@163.com (Z.M.); kz_chen@e.gzhu.edu.cn (K.C.); runtianyuan7316@163.com (R.Y.); lln1692768472@163.com (L.L.); 2 School of Intelligent Manufacturing and Automotive Technology, Guangzhou Huashang Vocational College, Guangzhou 511300, China; huangbixue@foxmail.com

**Keywords:** medicine–food homology, phytochemicals, geroscience, healthy aging, bioavailability, nanocarriers, telomere biology

## Abstract

Aging-related decline involves changes in oxidative stress, inflammation, mitochondrial function, telomere maintenance, metabolic regulation, and extracellular matrix homeostasis. This systematic review followed the Preferred Reporting Items for Systematic Reviews and Meta-Analyses (PRISMA) 2020 statement and searched PubMed, Scopus, Embase, and Web of Science for English-language studies published from 1993 to 2025. Twelve primary studies met the eligibility criteria: four randomized clinical studies, four animal studies, one in vitro mechanistic study, and three delivery-system/formulation studies. Risk of bias was assessed using Cochrane Risk of Bias 2 (RoB 2), the Systematic Review Centre for Laboratory Animal Experimentation (SYRCLE) tool, and a review-specific domain-based checklist informed by the National Toxicology Program/Office of Health Assessment and Translation framework. Representative compounds, including resveratrol, astragaloside IV, *Ganoderma lucidum* polysaccharide, epigallocatechin-3-gallate (EGCG) and betulinic acid, were associated with mitochondrial regulation, antioxidant defense, cytoskeletal stability, gut–brain signaling, extracellular-matrix protection, and longevity-related signaling. Nanoliposomes and poly(lactic-co-glycolic acid) (PLGA) nanoparticles improved selected stability, penetration, and pharmacokinetic outcomes. However, the evidence base was heterogeneous and predominantly preclinical; human evidence was limited to four relatively small studies, and certainty ranged from moderate to very low. The findings support mechanistic and translational potential but do not establish definitive anti-aging efficacy in humans.

## 1. Introduction

With the accelerating trend of global population aging, aging-related cardiovascular diseases, neurodegenerative disorders, and metabolic syndrome have become major public health challenges in the 21st century [1]. Against this backdrop, scientific and public attention has increasingly shifted from disease treatment alone toward prevention and the promotion of healthy aging. Nature-derived interventions, particularly bioactive compounds from medicinal and edible plants, have attracted growing research interest because of their potential to modulate multiple biological processes associated with aging [2]. Medicine–food homology materials also serve as traditional dietary resources and sources of chemically diverse bioactive constituents, supporting their investigation as ingredients for functional foods and nutraceuticals [3].

The original framework proposed nine hallmarks of aging in 2013. [1] An updated framework subsequently expanded this concept to 12 interconnected hallmarks by additionally recognizing disabled macroautophagy, chronic inflammation, and dysbiosis as distinct components of aging biology [4]. A growing body of evidence suggests that phytochemicals from medicinal and edible plants, including polyphenols, flavonoids, saponins, and polysaccharides, may modulate several of these interconnected aging-related processes through multi-target and multi-pathway mechanisms. Nuclear factor erythroid 2-related factor 2 (Nrf2) is a central regulator of endogenous antioxidant defense, and components such as curcumin may enhance cellular resistance to oxidative damage by activating Nrf2 signaling [5]. Persistent activation of nuclear factor kappa B (NF-κB) is associated with chronic inflammation, whereas astragaloside IV and *Ganoderma lucidum* polysaccharides have been reported to modulate NF-κB-related inflammatory signaling. The adenosine monophosphate-activated protein kinase (AMPK)/sirtuin 1 (SIRT1) pathway is an important energy-sensing cascade involved in mitochondrial function and metabolic homeostasis, with ginsenosides reported as potential regulators of this pathway [6]. Although existing studies have provided substantial evidence for the anti-aging potential of medicinal and edible plants, significant bottlenecks remain in this field: research evidence is highly fragmented, nano-delivery technology and molecular mechanism studies are disconnected from each other, the bioavailability of phytochemicals is insufficient due to low solubility and poor stability, and the translational gap from preclinical to clinical stages remains substantial, particularly because many delivery-system studies focus on physicochemical characterization rather than direct validation of aging-related molecular outcomes and long-term clinical efficacy [7,8]. These challenges are consistent with a recent review of flavonoids from medicine–food homology substances, which highlighted source- and processing-dependent compositional variability, difficulties in quality control, poor solubility, and low bioavailability as major barriers to practical application [9]. Moreover, regulatory frameworks for medicinal and edible products vary significantly across countries and regions, further complicating product standardization and market translation [10,11].

Several recent reviews have summarized the anti-aging potential of traditional plant-based foods, edible medicinal plants, medicine–food homology plants, and naturally derived bioactive compounds. Das et al. [12] reviewed the anti-aging effects of traditional plant-based foods, whereas Iweala et al. [13] summarized bioactive phytoconstituents from edible medicinal plants and their proposed mechanisms. Bjørklund et al. [14] provided a broader overview of natural compounds and products from an anti-aging perspective, and Zhu et al. [2] recently focused on plant-based anti-aging strategies involving medicine–food homology plants. These reviews establish an important foundation for the field. However, an integrated assessment that simultaneously distinguishes molecular plausibility, delivery-system performance, study-type-specific methodological quality, and the certainty of human clinical evidence remains valuable.

To address this evidence gap, the present systematic review was reported in accordance with the PRISMA 2020 statement and synthesized eligible in vitro, animal, delivery-system, and human intervention studies published between 1993 and 2025. Risk of bias was evaluated using Cochrane Risk of Bias 2 (RoB 2) for randomized clinical studies [15] and the Systematic Review Centre for Laboratory Animal Experimentation (SYRCLE) tool for animal studies [16]. Delivery-system/formulation studies were assessed using a review-specific domain-based checklist informed by the National Toxicology Program/Office of Health Assessment and Translation (NTP/OHAT) systematic-review framework [17]. Although non-randomized human intervention studies were eligible, none met the final inclusion criteria; therefore, the Risk Of Bias In Non-randomized Studies of Interventions (ROBINS-I) was not applied [18]. This review aimed to elucidate the principal molecular mechanisms associated with selected phytochemicals from medicinal and edible plants, evaluate delivery strategies intended to improve their stability and bioavailability, critically assess the strength of the available clinical evidence, and identify methodological priorities for future translational research.

## 2. Methods

### 2.1. Study Design and Search Strategy

**Figure 1:**
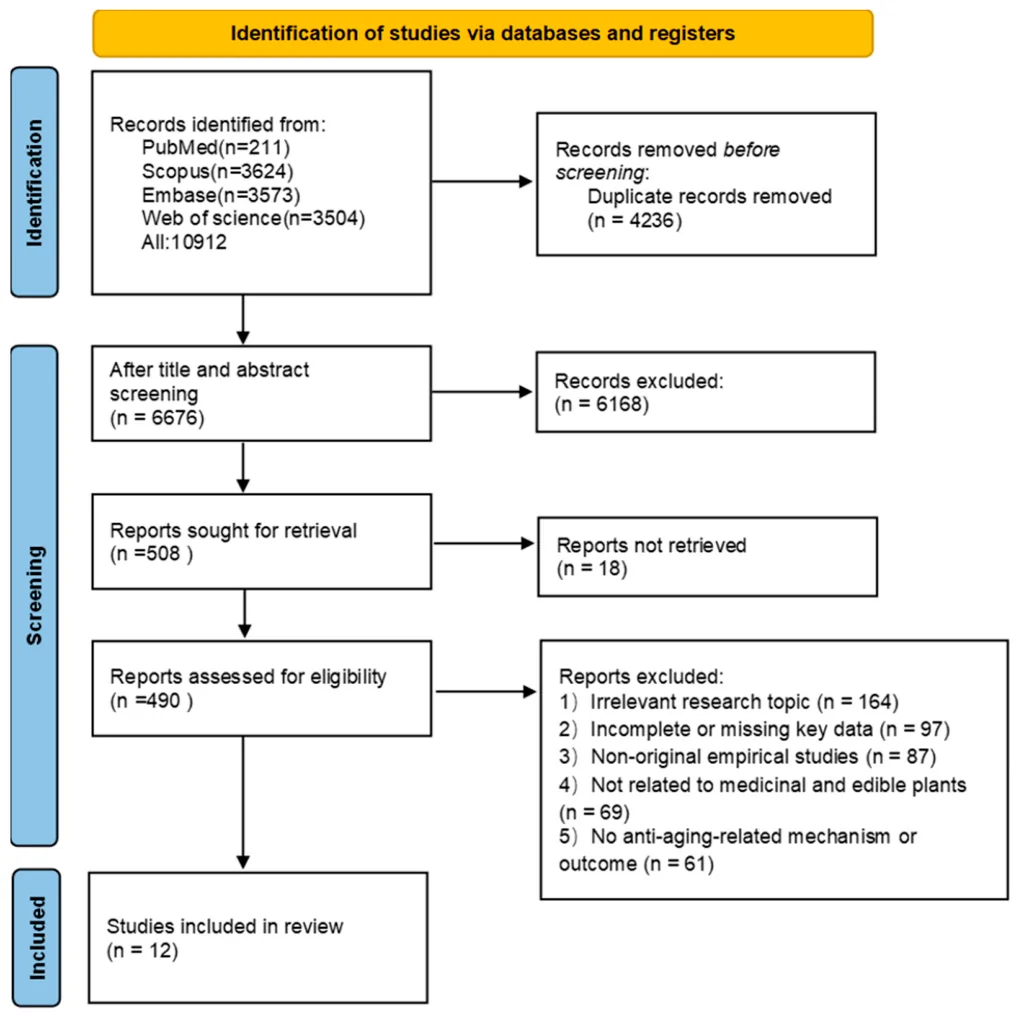
PRISMA 2020 flow diagram of the study-selection process. Searches of PubMed, Scopus, Embase, and Web of Science identified 10,912 records. After duplicate removal, title and abstract screening, full-text retrieval, and eligibility assessment, 12 primary studies were included: four randomized clinical studies, four animal studies, one standalone in vitro mechanistic study, and three delivery-system/formulation studies. Full-text exclusion reasons and corresponding numbers are presented in the diagram.

This systematic review was conducted in strict accordance with the Preferred Reporting Items for Systematic Reviews and Meta-Analyses (PRISMA) guidelines to ensure a transparent process and reproducible results [19,20,21]. The databases searched included PubMed/MEDLINE, Scopus, Web of Science Core Collection and EMBASE, covering the period from 1 January 1993 to 31 December 2025 [22,23,24]. The final search was performed using database-specific syntax in PubMed, Web of Science, Embase, and Scopus, with restrictions to English-language publications from 1993 to 2025. The detailed search strategies for each database are provided in Appendix A and Table A1. The completed PRISMA 2020 checklist is provided in Supplementary Table S1. The PRISMA flow diagram (Figure 1) was prepared using the official PRISMA 2020 Word template in Microsoft Word for Microsoft 365 (Microsoft Corporation, Redmond, WA, USA). Boolean operators were applied to construct search strategies, with core search dimensions including: medicinal and edible plants, phytochemicals, anti-aging, oxidative stress, inflammation, cellular senescence, signaling pathways, nano-delivery systems, and clinical trials. The search strategy was adapted according to the syntax of each database, and supplementary searches were performed for key plants and components such as Panax ginseng, *Ganoderma lucidum*, curcumin, resveratrol, and epigallocatechin gallate [13,21]. Additionally, reference lists of included articles and relevant reviews were manually screened to further improve search completeness. Selected reviews and standards published in 2026 were used only to contextualize recent developments in the Introduction and Discussion; they were not considered for study eligibility, data extraction, risk-of-bias assessment, or the PRISMA flow.

### 2.2. Inclusion and Exclusion Criteria

Inclusion criteria: (1) Study types included in vitro studies, animal experiments, and human clinical trials, including randomized controlled trials and non-randomized intervention studies; (2) Research subjects were extracts, fractions, or single active ingredients derived from medicinal and edible plants; (3) The studies clearly reported anti-aging effects, molecular mechanisms, delivery systems, or clinical intervention outcomes; (4) In vitro studies used aging-related cell models, animal studies used recognized aging models, and clinical studies included appropriate controls; (5) Peer-reviewed English literature published between 1993 and 2025 [13].

Exclusion criteria: (1) Non-original empirical studies such as reviews, meta-analyses, conference abstracts, patents, or dissertations; (2) Synthetic compounds of non-plant origin or irrelevant natural products; (3) Studies with unavailable full text, missing key data, or unclear presentation of results.

### 2.3. Literature Screening and Data Extraction

All retrieved records were imported into EndNote 2025 (Clarivate, London, UK) for reference management, duplicate removal, and organization of the screening process. Two reviewers independently screened all deduplicated records in two sequential stages. Titles and abstracts were first assessed against the prespecified eligibility criteria, after which potentially eligible reports were evaluated through full-text review. Screening decisions were compared at each stage. Disagreements were resolved through discussion, with unresolved cases adjudicated by a third reviewer. Final eligibility decisions were reached by consensus. No formal statistical measure of inter-reviewer agreement, such as Cohen’s kappa coefficient, was calculated. A standardized data-extraction form was developed in Microsoft Excel for Microsoft 365 (Microsoft Corporation, Redmond, WA, USA), and two reviewers independently extracted information on the first author, publication year, botanical name, plant part used, bioactive compound, study model, intervention protocol, control or comparator, primary outcomes, molecular mechanisms, safety data, and principal conclusions.

**Figure 2:**
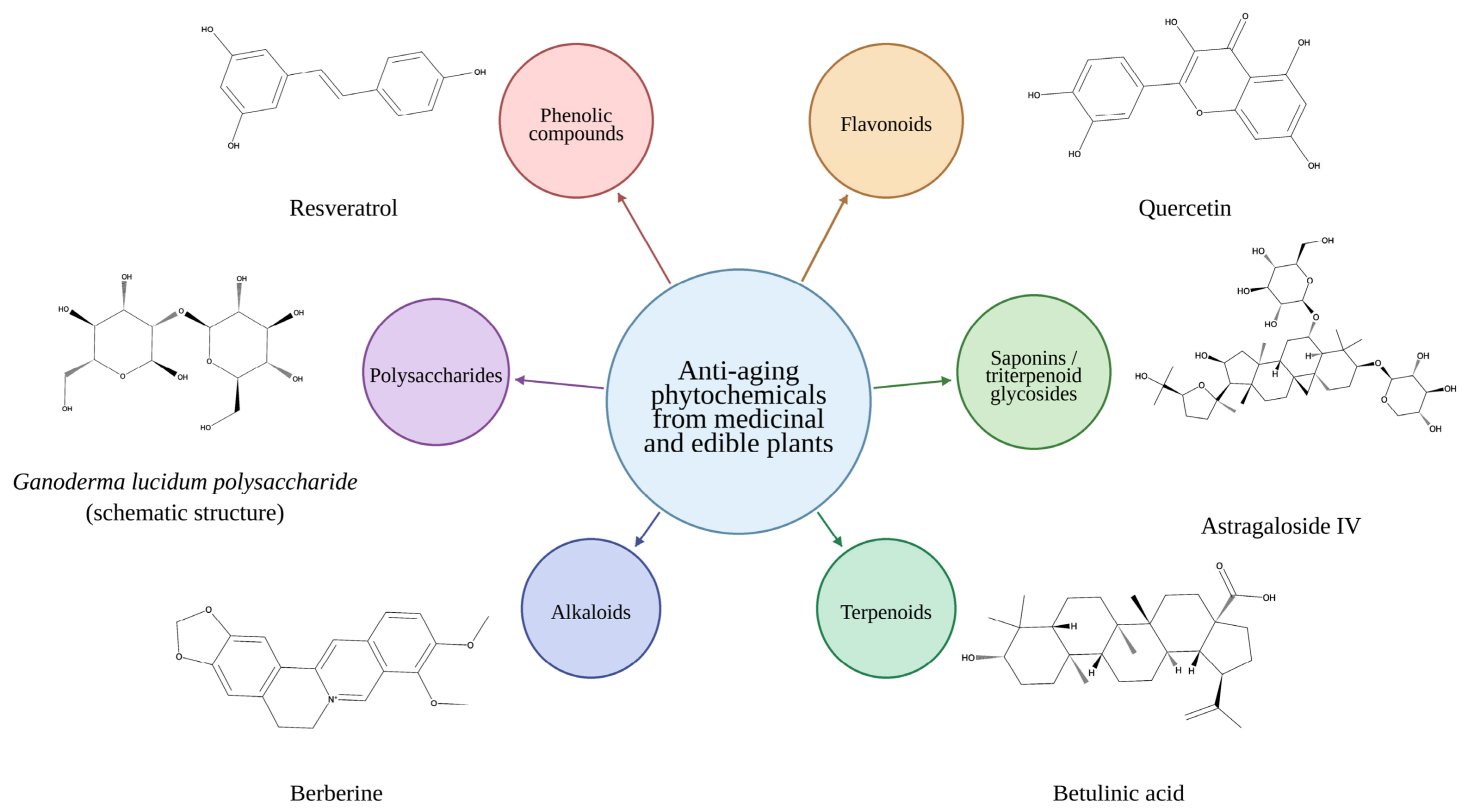
Representative structural classes and selected chemical structures of bioactive compounds from medicinal and edible plants.

**Figure 3:**
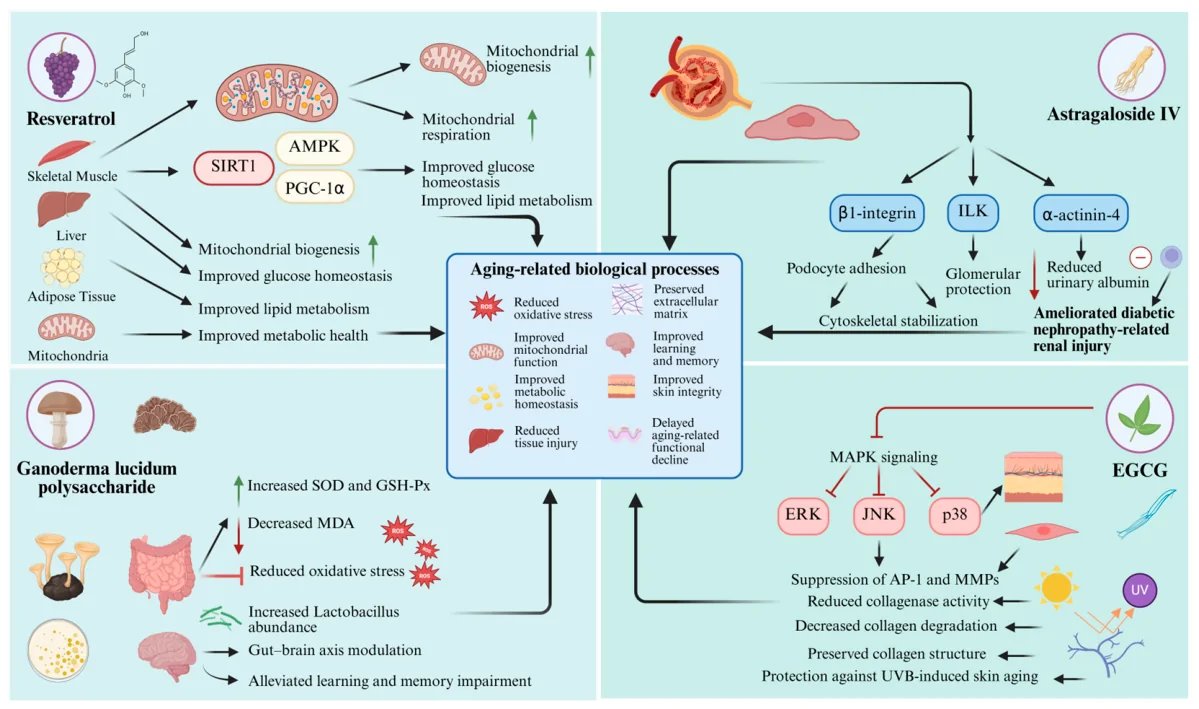
Representative aging-related molecular mechanisms associated with selected bioactive compounds from medicinal and edible sources.

**Figure 4:**
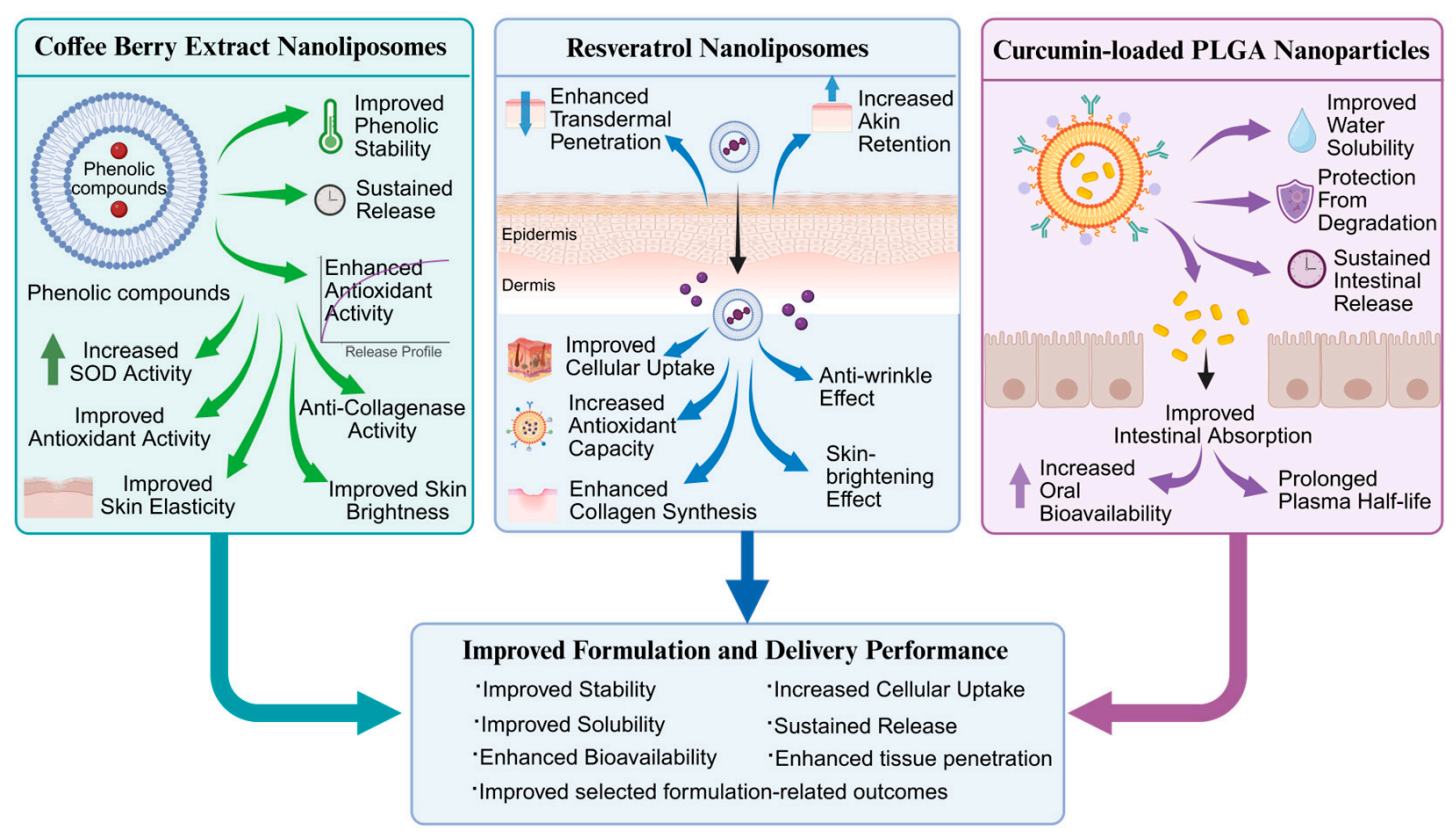
Delivery strategies used to improve the physicochemical and pharmacokinetic performance of selected bioactive compounds.

Each included study was assigned to one mutually exclusive category according to its primary research objective: randomized clinical study, animal study, in vitro mechanistic study, or delivery-system/formulation study. Studies incorporating multiple experimental components were classified according to their principal objective and were counted only once. For example, formulation studies incorporating in vitro assays, three-dimensional skin models, or animal pharmacokinetic experiments were classified as delivery-system studies. Figure 2, Figure 3 and Figure 4 were created using the BioRender web-based platform (Science Suite Inc., Toronto, ON, Canada).

### 2.4. Risk of Bias Assessment

Risk of bias was assessed using study-type-specific tools. The Cochrane RoB 2 tool [15] was used for randomized clinical studies, and the SYRCLE risk-of-bias tool was used for animal studies. Although non-randomized controlled human intervention studies were eligible according to the inclusion criteria, no such study met the final eligibility criteria; therefore, ROBINS-I [18] was not applied.

For delivery-system/formulation studies, risk of bias was assessed using a review-specific domain-based checklist informed by the National Toxicology Program/Office of Health Assessment and Translation (NTP/OHAT) systematic-review framework [17]. This approach was selected because the included studies comprised heterogeneous experimental designs, including in vitro assays, three-dimensional skin models, in vivo skin evaluations, and animal pharmacokinetic experiments, which were not fully covered by a single standard design-specific tool. The checklist evaluated eight domains: adequacy of dose or exposure administration, group allocation, comparability of experimental conditions, completeness of outcome data, exposure or formulation characterization, assessor blinding, completeness of outcome reporting, and other potential threats to internal validity. Each domain was judged as having low, unclear, or high risk of bias. Low risk indicated that the reported methods adequately minimized the relevant source of bias; unclear risk indicated that insufficient information was available to support a definitive judgment; and high risk indicated an identifiable methodological limitation likely to materially affect internal validity. One reviewer performed the initial assessment, and a second reviewer independently verified all judgments. Disagreements were resolved through discussion. Judgments were made separately for each domain, and no numerical total score was calculated. Detailed operational criteria are provided in Supplementary Table S3. The single standalone in vitro mechanistic study was not subjected to a formal risk-of-bias tool because no validated tool specifically applicable to this study design had been prespecified. Its methodological limitations were considered narratively when interpreting the evidence. The formal risk-of-bias assessments and narrative appraisal were used to interpret the methodological robustness of the randomized clinical, animal, delivery-system/formulation, and standalone in vitro evidence.

Risk-of-bias assessments for the randomized clinical studies were completed using the official RoB 2 Excel tool (version 22 August 2019; University of Bristol, Bristol, UK). Risk-of-bias summary figures for the randomized clinical, animal, and delivery-system/formulation studies were generated using Review Manager 5 (RevMan 5), version 5.4 (The Cochrane Collaboration, Copenhagen, Denmark).

### 2.5. Certainty of Evidence Assessment

The certainty of evidence for clinical outcomes was assessed using the Grading of Recommendations Assessment, Development and Evaluation (GRADE) approach. Only randomized clinical studies with human participants were included in the GRADE assessment. The certainty of evidence was evaluated across five domains: risk of bias, inconsistency, indirectness, imprecision, and publication bias. Randomized clinical trials started as high-certainty evidence and were downgraded when serious or very serious limitations were identified. Animal studies, mechanistic studies, and delivery-system studies were not included in the main GRADE evidence table but were summarized separately as preclinical and translational evidence.

## 3. Results

### 3.1. Literature Screening Results

A total of 10,912 records were identified through database searches, including 211 from PubMed, 3504 from Web of Science, 3573 from Embase, and 3624 from Scopus. After removing 4236 duplicate records, 6676 records remained for title and abstract screening. Following screening, 6168 records were excluded, and 508 reports were sought for full-text retrieval. Eighteen reports could not be retrieved, leaving 490 reports for full-text eligibility assessment. After full-text assessment, 478 reports were excluded for the following reasons: irrelevant research topic (*n* = 164), incomplete or missing key data (*n* = 97), non-original empirical studies (*n* = 87), not related to medicinal and edible plants (*n* = 69), and no anti-aging-related mechanism or outcome (*n* = 61). Finally, 12 studies were included in the systematic review. The literature screening process is shown in Figure 1.

### 3.2. Basic Characteristics of Included Studies

A total of 12 primary studies met the eligibility criteria. According to their principal research objectives, these comprised four randomized clinical studies [17,18,19,20], four animal studies [21,22,23,25], one in vitro mechanistic study [26], and three delivery-system/formulation studies [5,6,24]. Each study was assigned to one mutually exclusive category and was counted only once. All four eligible human intervention studies were randomized clinical studies, and no eligible non-randomized human intervention study was identified. Complete study-level characteristics and classifications are provided in Supplementary Table S2.

The included studies investigated a range of medicinal and edible plants and their bioactive compounds. The four randomized clinical studies summarized in Table 1 evaluated TA-65 and resveratrol in healthy adults, individuals with obesity, and patients with type 2 diabetes mellitus [17,18,19,20]. The four animal studies summarized in Table 2 investigated resveratrol in high-fat diet-induced obese mice, astragaloside IV in rats with diabetic nephropathy, Ganoderma lucidum polysaccharides in D-galactose-induced aging mice, and betulinic acid in Drosophila melanogaster [21,22,23,25]. The single standalone in vitro mechanistic study examined the effects of epigallocatechin-3-gallate on ultraviolet B-induced collagen degradation in human dermal fibroblasts [26]. Table 3 summarizes the principal molecular mechanisms identified across the included evidence, including the SIRT1/PGC-1α and AMPK/SIRT1/PGC-1α pathways associated with resveratrol, β1-integrin/ILK signaling and α-actinin-4-mediated podocyte cytoskeletal regulation associated with astragaloside IV, antioxidant defense and gut–brain axis regulation associated with G. lucidum polysaccharides, and MAPK-mediated MMP/collagen degradation regulation associated with epigallocatechin-3-gallate [20,21,22,23,26]. Table 3 therefore represents a cross-study mechanistic synthesis rather than an additional category of included studies. The three delivery-system studies summarized in Table 4 investigated coffee berry extract nanoliposomes, resveratrol nanoliposomes, and curcumin-loaded poly(lactic-co-glycolic acid) nanoparticles [5,6,24]. The methodological quality and certainty of the included evidence were assessed according to study type, as described in Section 3.4.

### 3.3. Anti-Aging Evidence of Phytochemicals

The anti-aging active ingredients in medicinal and edible plants are diverse and can be broadly classified based on their chemical skeletons into phenolic compounds, terpenoids, saponins, alkaloids, polysaccharides, and other structural classes [14,35]. Understanding the chemical structures of these compounds is fundamental to elucidating their structure–activity relationships and multi-target mechanisms of action [35]. Phenolic compounds represent a major group of food-derived phytochemicals and include flavonoids, phenolic acids, stilbenes, lignans, and tannins, which are widely found in tea, grapes, berries, fruits, vegetables and other plant-derived foods [13]. Terpenoids are biosynthetically derived from isoprene units and comprise structurally diverse subclasses, including monoterpenoids, sesquiterpenoids, diterpenoids, triterpenoids, and tetraterpenoids [13]. Representative medicinal and edible sources of terpenoids include *Panax ginseng*, citrus species, and the medicinal fungus *Ganoderma lucidum* [14]. Saponins generally consist of a hydrophobic aglycone linked through glycosidic bonds to one or more hydrophilic sugar moieties, with ginsenosides such as Rb1 and Rg1 representing well-known examples from *P. ginseng* [36]. Alkaloids are structurally diverse nitrogen-containing natural products, among which berberine is a representative compound found in several medicinal plants [13]. Polysaccharides, including *G. lucidum* polysaccharides and *Lycium barbarum* polysaccharides, are macromolecules composed of monosaccharide units linked by glycosidic bonds and have been widely investigated as bioactive constituents of medicinal and edible materials [3]. Representative structural classes and selected chemical structures of major anti-aging phytochemicals from medicinal and edible plants are shown in Figure 2. Representative molecular mechanisms and delivery-system strategies are further summarized in Table 3 and Table 4 and illustrated in Figure 3 and Figure 4.

#### 3.3.1. Evidence at the Molecular Mechanism Level

Active ingredients such as polyphenols, flavonoids, saponins, and polysaccharides from medicinal and edible plants can exert anti-aging effects by regulating multiple aging-related signaling pathways. Broadly, natural food-derived compounds have been reported to modulate antioxidant, inflammatory, metabolic, and extracellular matrix remodeling pathways, including Nrf2/ARE, NF-κB, AMPK/SIRT1, and MAPK-related signaling [35]. Among the representative studies summarized in Table 3, resveratrol is one of the most extensively studied stilbenoids and can activate the SIRT1/PGC-1α axis and AMPK/SIRT1/PGC-1α signaling, thereby enhancing mitochondrial biogenesis, improving mitochondrial respiration, and regulating metabolic homeostasis [25,27]. Astragaloside IV showed protective effects in diabetic nephropathy models by regulating β1-integrin, integrin-linked kinase, and α-actinin-4, suggesting a role in podocyte adhesion and cytoskeletal stabilization [30]. *Ganoderma lucidum* polysaccharide improved learning and memory impairment in D-galactose-induced aging mice by enhancing antioxidant defense, increasing SOD and GSH-Px activities, reducing MDA levels, and modulating gut microbiota, especially *Lactobacillus* abundance [31]. EGCG can reduce collagen destruction and collagenase activation in UVB-irradiated human dermal fibroblasts by inhibiting MAPK-related signaling and suppressing MMP production [33,37]. In addition, other natural compounds such as betulinic acid may regulate longevity-related Sir2/FOXO signaling in model organisms [32]. Collectively, these components exhibit anti-aging characteristics involving multi-target and multi-pathway regulation.

Based on the representative mechanisms summarized above, Figure 3 illustrates the integrated molecular network through which medicinal and edible plant-derived compounds exert anti-aging effects.

#### 3.3.2. Evidence from Delivery System Studies

Nano-delivery technologies can effectively address the key bottlenecks of poor solubility, low stability, insufficient skin penetration, and limited bioavailability of phytochemicals. Coffee berry extract nanoliposomes improved phenolic stability and sustained release, enhanced SOD activity and anti-collagenase activity, and improved skin elasticity and brightness in human skin [7]. Resveratrol nanoliposomes improved transdermal penetration and skin retention, enhanced cellular uptake, promoted antioxidant capacity and collagen synthesis, inhibited MMP secretion, and showed anti-wrinkle and skin-brightening effects [8]. Curcumin-loaded PLGA nanoparticles improved water solubility, showed sustained intestinal release, increased oral relative bioavailability, and prolonged plasma half-life compared with native curcumin [34]. In addition, piperine can enhance the absorption efficiency of curcumin and other phytochemicals by inhibiting metabolic enzymes and efflux transporters [38,39], while fermentation treatments and composite nanocarriers may further optimize component stability and enhance biological efficacy.

The major delivery strategies for enhancing the anti-aging efficacy of plant-derived bioactives are summarized in Figure 4.

#### 3.3.3. Evidence from Preclinical Studies

In vitro cell models and animal experiments consistently confirm that active ingredients from medicinal and edible plants possess significant anti-aging effects. Resveratrol improved mitochondrial function and exercise endurance in high-fat diet-fed mice by activating the SIRT1/PGC-1α pathway [25]. Astragaloside IV improved renal function in streptozotocin-induced diabetic nephropathy rats by reducing blood glucose and urinary microalbumin, improving renal pathological changes, and regulating podocyte adhesion-related proteins, including β1-integrin, ILK, and α-actinin-4 [30]. *Ganoderma lucidum* polysaccharide improved learning and memory impairment in D-galactose-induced aging mice by enhancing antioxidant capacity, reducing lipid peroxidation and oxidative stress, and modulating intestinal flora [31]. EGCG inhibited UVB-induced ASK-1 activation and phosphorylation of JNK, p38 MAPK, and ERK1/2, thereby suppressing MMP production and reducing collagen destruction in human dermal fibroblasts [33]. In addition, betulinic acid has been reported to extend the lifespan of Drosophila in a Sir2/FOXO-dependent manner [32]. Overall, preclinical studies provide mechanistic evidence for the anti-aging effects of plant-derived active ingredients.

#### 3.3.4. Evidence from Clinical Studies

This review included four randomized clinical studies, and the results showed that plant-derived active ingredients have positive but still limited anti-aging-related effects. TA-65, as an *Astragalus membranaceus*-derived telomerase activator, significantly prolonged leukocyte telomere length in healthy individuals after 12 months of intervention [26]. Resveratrol supplementation reduced resting metabolic rate, systolic blood pressure, blood glucose, triglycerides, and liver lipids in obese individuals [27]. In patients with type 2 diabetes mellitus, Bhatt et al. [28] reported that resveratrol supplementation improved HbA1c and several cardiometabolic parameters, whereas Brasnyo et al. [29] reported decreased HOMA-IR and urinary ortho-tyrosine excretion and increased platelet pAkt/Akt ratio. However, the included clinical studies generally had small sample sizes and relatively short intervention durations. Large-scale, long-term, multicenter randomized controlled trials remain scarce, and clinical evidence still requires further expansion and validation.

### 3.4. Risk of Bias and Certainty of Evidence Assessment

**Figure 5:**
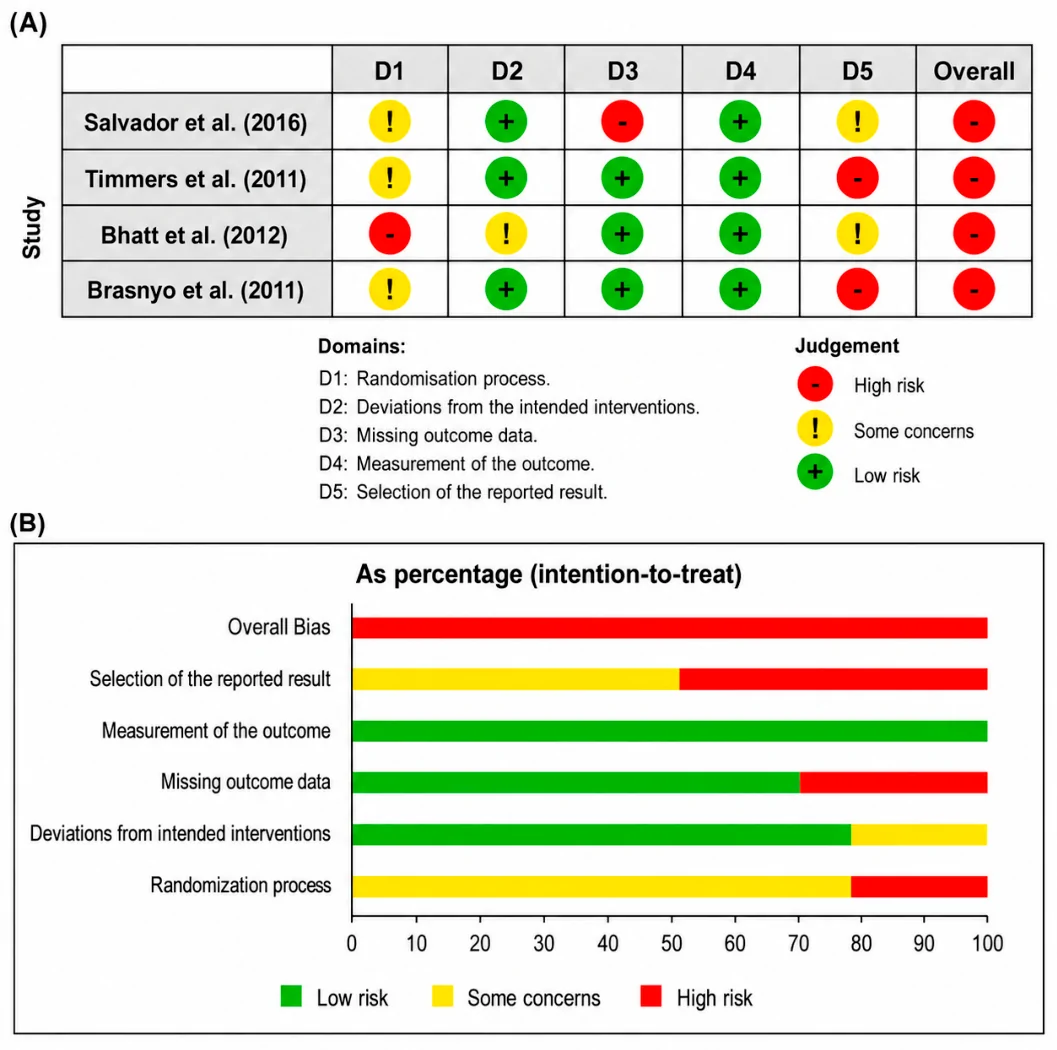
Risk-of-bias assessment of included randomized clinical trials using the Cochrane RoB 2 tool: (**A**) risk-of-bias summary; (**B**) percentage summary of risk-of-bias judgments. The included trials were Salvador et al. [26], Timmers et al. [27], Bhatt et al. [28], and Brasnyó et al. [29].

Risk of bias and certainty of evidence were assessed according to study type. For randomized clinical studies, the Cochrane RoB 2 tool was used to evaluate bias across five domains, including the randomization process, deviations from intended interventions, missing outcome data, outcome measurement, and selection of the reported result. The domain-level judgments and overall risk-of-bias assessments are summarized in Table 5, and the corresponding risk-of-bias summary and percentage distribution are shown in Figure 5. Because randomized clinical studies constituted the available source of human intervention evidence, the certainty of anti-aging-related clinical outcomes was further evaluated using the GRADE approach, as shown in Table 6.

**Figure A1:**
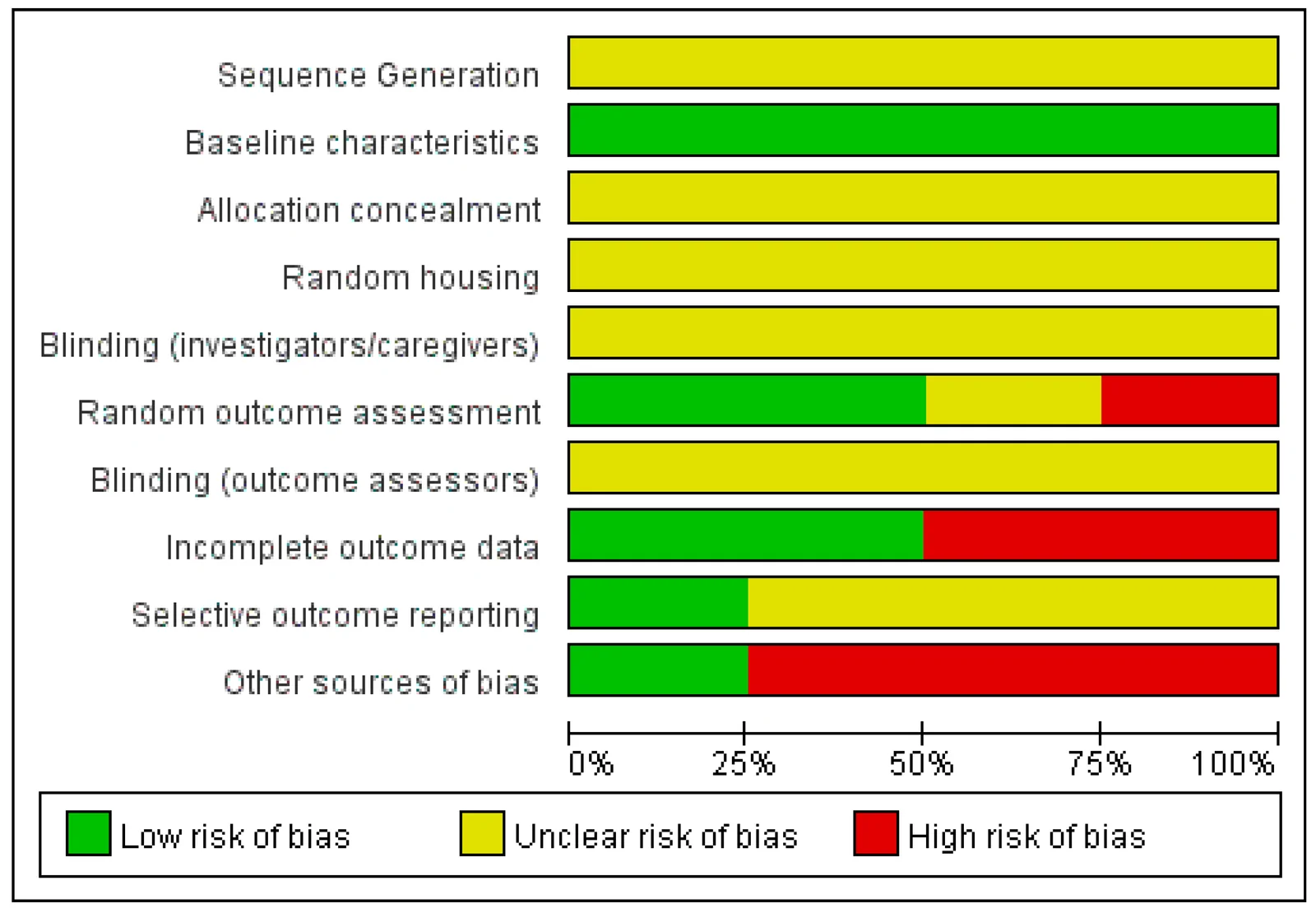
Risk-of-bias judgments for the included animal studies assessed using the SYRCLE tool.

For the four included animal studies, risk of bias was assessed using the SYRCLE tool. Baseline characteristics were judged to be at low risk of bias in all four studies. However, sequence generation, allocation concealment, random housing, blinding of investigators or caregivers, and blinding of outcome assessors were generally judged as unclear because the corresponding procedures were insufficiently reported. High-risk judgments were identified for incomplete outcome data in Lu et al. [30] and Chen et al. [31], for random outcome assessment in Chen et al. [31], and for other potential sources of bias in Lagouge et al. [25], Chen et al. [31], and Lee et al. [32]. The study-level judgments are presented in Table 7, and the corresponding graphical summary is provided in Appendix B and Figure A1.

**Figure A2:**
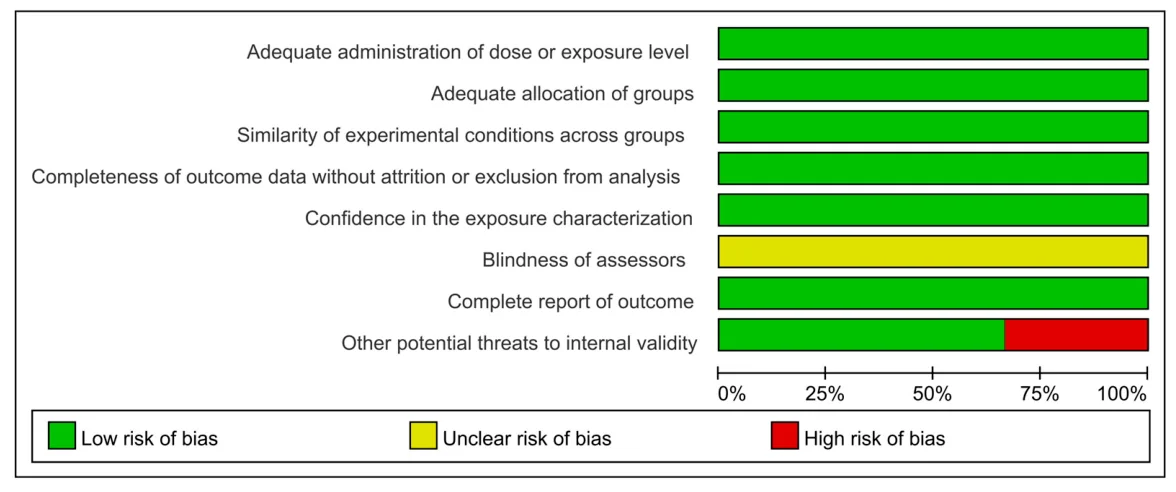
Summary of risk-of-bias judgments for the included delivery-system/formulation studies assessed using a review-specific domain-based checklist informed by the NTP/OHAT systematic-review framework.

The three delivery-system/formulation studies were evaluated using the review-specific domain-based checklist described in Section 2.4. All three studies were judged to be at low risk of bias regarding dose or exposure administration, group allocation, comparability of experimental conditions, completeness of outcome data, exposure of formulation characterization, and completeness of outcome reporting. Assessor blinding was judged as unclear in all three studies because the relevant procedures were not adequately reported. Other potential sources of bias were judged to be high for Zhang et al. [8] and low in Saewan et al. [7] and Xie et al. [34]. The study-level judgments are summarized in Table 8, and the corresponding graphical presentation is provided in Appendix B (Figure A2). The single standalone in vitro mechanistic study by Bae et al. [33] was not included in the formal risk-of-bias tables or figures because no validated tool specifically applicable to this study design had been prespecified. Its methodological limitations, including model specificity, exposure conditions, outcome selection, and reporting completeness, were considered narratively when interpreting the findings.

### 3.5. Safety, Quality Control, and Regulatory Status

The safety of medicinal and edible plants is a prerequisite for their promotion and application as functional foods. The case of licorice (*Glycyrrhiza glabra* L.) typifies the dose-dependent dual role of phytochemicals. A large number of animal experiments and in vitro studies have confirmed that licorice extract and its main active ingredients, glycyrrhizic acid and glycyrrhetinic acid, can effectively counteract chemical- or alcohol-induced liver injury through antioxidant, anti-inflammatory, and anti-fibrotic mechanisms [40]. In clinical practice, licorice preparations are used as adjunctive therapy for liver diseases such as viral hepatitis [40]. However, long-term or high-dose intake of licorice poses significant health risks, primarily inducing pseudoaldosteronism, which leads to hypertension, hypokalemia, and edema [41,42]. The mechanism involves the inhibition of renal 11β-hydroxysteroid dehydrogenase type 2 by glycyrrhizin metabolites, causing cortisol to persistently activate the mineralocorticoid receptor, resulting in sodium and water retention and elevated blood pressure [43,44]. This risk shows a clear dose-dependent relationship [45,46], but clinical trials specifically designed to precisely quantify the dose–response curve comparing the hepatoprotective effects and hypertension risk of licorice are still lacking [47,48].

Regarding quality control, pharmacopoeial and international standards provide an important basis for the authentication and consistency of *Ganoderma lucidum* products. The 2020 edition of the Chinese Pharmacopoeia includes *G. lucidum* and its spores and specifies that *G. lucidum* spore powder should contain not less than 0.90% polysaccharides, calculated as anhydrous glucose, and not less than 0.50% total triterpenes and sterols, calculated as oleanolic acid [49]. The United States Pharmacopeia also provides monographs for *G. lucidum*-related materials [49,50]. The second edition of ISO 21315, published in 2026, specifies requirements and test methods for *G. lucidum* fruiting bodies used as Chinese materia medica [51]. However, the chemical composition and quality of *Ganoderma* materials can vary with species, cultivation conditions, cultivation substrates, processing methods, and storage duration [49,50]. A recent review of *Ganoderma* systematics, phylogeny, and metabolomics further supports combining molecular authentication with chromatographic or metabolomic fingerprinting to improve species discrimination and product standardization [52]. Moreover, quality assessment based on only a small number of marker compounds may not adequately represent the complex chemical profile, interactions among constituents, or biological potency of the final product [50].

Adulteration is a persistent and pervasive problem in the herbal and dietary supplement industry. Studies worldwide indicate that up to 27% of herbal products are adulterated, containing undeclared substitutes, contaminants, or fillers [53]. In a specific case involving *Ganoderma lucidum* products, dietary supplements marketed in the United States were found to contain starch-based substances, a typical example of economically motivated adulteration [54]. A more subtle form of adulteration involves species substitution; products labeled as *Ganoderma lucidum* may contain other, less expensive but morphologically similar *Ganoderma* species or even entirely unrelated fungi [55]. For high-value products such as *Ganoderma lucidum* spore oil, dilution with cheap vegetable oils like rapeseed oil or sunflower seed oil is a common adulteration practice [56]. Headspace-gas chromatography-ion mobility spectrometry has been proven to rapidly and accurately identify adulterated vegetable oils in *Ganoderma lucidum* spore oil, achieving effective discrimination even at adulteration levels as low as 5% [56].

Regarding sustainable production methods, plant cell culture technology is applied to the production of plant-derived secondary metabolites. Large-scale bioreactor cultivation of suspension cells of *Taxus chinensis* (Pilg.) Rehder enables the industrial production of paclitaxel (Taxol^®^) [57]. This plant-cell culture approach provides sterile and controllable production conditions, is less dependent on geographical and seasonal constraints, facilitates downstream purification, and reduces the risk of pesticide and heavy-metal contamination [58]. Mycelial submerged fermentation technology is currently the most efficient and mainstream industrial production method for fungi such as *Ganoderma lucidum*. By providing optimized liquid culture media for *G. lucidum* mycelia in large-scale stainless steel bioreactors, large quantities of standardized mycelial biomass and active substances such as extracellular polysaccharides can be obtained within weeks [59,60]. Simulation analyses indicate that the production cost of *G. lucidum* fermentation is closely related to reactor volume; when the bioreactor volume is increased from 2 m^3^ to 200 m^3^, the unit production cost can drop dramatically from approximately $6.82/g to $0.17/g [61]. The industry has already demonstrated the feasibility of commercial production, achieving yields of 15 kg of dried mycelium from 300 L of culture medium in a single fermentation cycle of about 30 days [62].

## 4. Discussion

The present systematic review identified 12 eligible primary studies, comprising four randomized clinical studies, four animal studies, one standalone in vitro mechanistic study, and three delivery-system/formulation studies [7,8,25,26,27,28,29,30,31,32,33,34]. The resulting evidence base was small, heterogeneous, and predominantly preclinical. Collectively, the included studies suggest that selected bioactive compounds from medicinal and edible sources may modulate mitochondrial regulation, antioxidant defense, metabolic homeostasis, cytoskeletal stability, gut–brain signaling, and extracellular-matrix remodeling. Earlier reviews summarized broad ranges of naturally derived anti-aging compounds [14,35]. More recent reviews have focused on medicine–food homology plants [2], traditional plant-based foods [12], edible medicinal plants [13], and the bioactive constituents and applications of medicine–food homology substances [3]. In contrast, the present review integrates molecular mechanisms, delivery-system performance, human clinical evidence, and study-type-specific methodological appraisal within a single evidence framework. This approach helps distinguish mechanistic plausibility and formulation-related improvements from evidence supporting clinically meaningful anti-aging effects. However, because only four randomized clinical studies were identified, the findings should not be interpreted as demonstrating class-wide or definitive anti-aging efficacy in humans.

The mechanistic findings can be interpreted within the updated framework of 12 interconnected hallmarks of aging, which include mitochondrial dysfunction, deregulated nutrient sensing, chronic inflammation, loss of proteostasis, disabled macroautophagy, and dysbiosis [4]. A recent synthesis of dietary bioactive compounds and inflammaging indicates that polyphenols such as resveratrol, EGCG, curcumin, and quercetin converge on NF-κB, Nrf2, and sirtuin-related pathways [63]. This broader evidence is consistent with the convergence of stress-response, inflammatory, and metabolic pathways observed across the representative studies included in the present review. Resveratrol was associated with sirtuin 1/peroxisome proliferator-activated receptor gamma coactivator 1-alpha and adenosine monophosphate-activated protein kinase/sirtuin 1/peroxisome proliferator-activated receptor gamma coactivator 1-alpha signaling in metabolic models [25,27]. Astragaloside IV regulated β1-integrin/integrin-linked kinase signaling and α-actinin-4-related podocyte cytoskeletal stability in diabetic nephropathy [30]. *Ganoderma lucidum* polysaccharides improved antioxidant indicators, learning and memory, and gut-microbiota composition in D-galactose-treated mice [31]. These model-specific findings can be placed within a broader microbiome–aging framework, as contemporary research indicates that age-related dysbiosis may influence aging trajectories through gut–organ axes, immune regulation, intestinal-barrier integrity, and microbially derived metabolites [64]. Epigallocatechin-3-gallate reduced mitogen-activated protein kinase-related matrix metalloproteinase activation and collagen degradation in ultraviolet B-exposed dermal fibroblasts [33]. Betulinic acid extended lifespan in *Drosophila melanogaster* through silent information regulator 2- and forkhead box O-dependent mechanisms [32]. These findings indicate convergence across stress-response, metabolic, and tissue-maintenance pathways, but they should not be interpreted as evidence of a single unified anti-aging mechanism. The included models represent distinct biological contexts: diabetic nephropathy reflects disease-related renal injury, ultraviolet B exposure primarily models localized photoaging, high-fat-diet models predominantly reflect metabolic dysfunction, and lifespan extension in fruit flies may not be directly extrapolated to mammalian or human aging. Moreover, differences in compounds, doses, administration routes, experimental durations, and outcome definitions prevent direct comparison of pathway-level effects across studies. The mechanistic evidence therefore supports hypothesis generation and biological plausibility rather than confirmation of generalized organismal anti-aging activity.

The three delivery-system/formulation studies provide preliminary evidence that nanoformulation may address selected physicochemical and pharmacokinetic limitations of phytochemicals [7,8,34]. Coffee berry extract and resveratrol nanoliposomes improved stability, release behavior, skin penetration or retention, and selected skin-related outcomes, whereas curcumin-loaded poly(lactic-co-glycolic acid) nanoparticles increased aqueous solubility, intestinal release, oral bioavailability, and plasma exposure. Recent work on resveratrol nanodelivery similarly highlights improvements in stability and delivery together with unresolved concerns regarding toxicity and targeted release [65]. Complementing these findings, a 2026 review of nanoencapsulation in food systems emphasized that delivery systems should be evaluated not only in terms of improved stability and bioavailability but also with respect to gastrointestinal fate, industrial scalability, safety, and regulatory feasibility [66]. At the food-product level, a recent Foods review further emphasized that the functional performance of encapsulated bioactives should be evaluated throughout food processing, storage, gastrointestinal digestion, and controlled release, including their post-digestion bioactivity in realistic food matrices [67]. Importantly, improvements in particle size, encapsulation efficiency, zeta potential, skin retention, or plasma exposure are intermediate formulation outcomes and do not by themselves demonstrate delayed aging or clinically meaningful anti-aging efficacy. None of the included studies established a quantitative relationship linking nanocarrier characteristics to the magnitude of pathway regulation or long-term aging-related outcomes. The studies also differed substantially in administration route, experimental model, carrier composition, and outcome measurement, limiting cross-study comparison. The risk-of-bias assessment identified unclear reporting of assessor blinding in all three delivery-system/formulation studies (Table 8 and Figure A2). Translation into functional foods will therefore require characterization under realistic gastrointestinal or dermal conditions, evaluation of degradation and tissue exposure, long-term safety assessment, industrial scalability, batch reproducibility, and compliance with regulatory requirements for small particles and nanomaterials in food products [66,68].

Human evidence was limited to four randomized studies evaluating TA-65 or resveratrol in markedly different populations, including healthy adults, individuals with obesity, and patients with type 2 diabetes mellitus [26,27,28,29]. Intervention durations, doses, and outcomes varied substantially, and the assessed outcomes ranged from leukocyte telomere length to glycemic, lipid, insulin-signaling, and oxidative-stress-related biomarkers. Such heterogeneity, together with the small number of studies, precluded quantitative synthesis. The Grading of Recommendations Assessment, Development and Evaluation assessment indicated moderate certainty for leukocyte telomere length but low or very low certainty for most metabolic, oxidative-stress-related, and mechanistic outcomes (Table 6). Although these studies provide preliminary signals of biological activity, telomere length, homeostatic model assessment of insulin resistance, platelet phosphorylated Akt-to-total Akt ratios, and oxidative-stress biomarkers should not automatically be interpreted as validated surrogate measures of clinically meaningful slowing of aging. Geroscience trial frameworks emphasize that biomarker changes should be linked to functional capacity, age-related disease burden, patient-reported outcomes, or other patient-important health outcomes before being accepted as evidence of efficacy [69]. A recent expert consensus further recommends evaluating complementary physiological, inflammatory, functional, and epigenetic biomarker domains rather than relying on a single aging-related marker [70]. Recent recommendations from the Intrinsic Capacity, Frailty and Sarcopenia Research Task Force similarly call for harmonized minimum datasets and for aging biomarkers to be validated against frailty, intrinsic capacity, and other clinically relevant outcomes [71]. A recent synthesis of dietary bioactive compounds and inflammaging further concluded that human evidence remains focused largely on biological markers and healthspan-related outcomes rather than demonstrated lifespan extension. [63]. Against this background, the four studies do not establish whether the observed effects are sustained, applicable to older or frail populations, or associated with reduced morbidity, improved physical or cognitive function, or prolonged healthspan. The human findings should therefore be regarded as preliminary and compound-specific rather than as evidence supporting general recommendations for medicinal and edible plant-derived anti-aging interventions. This cautious interpretation is consistent with a recent review of dietary polyphenols in brain aging, which concluded that mechanistic evidence remains predominantly preclinical and that clinical translation is constrained by heterogeneity in intervention composition, dose, duration, and outcome measurement [72]. Translation into anti-aging functional foods also depends on ingredient identity, dose, product consistency, and long-term safety. Licorice illustrates why biological activity cannot be considered separately from exposure: licorice extracts and glycyrrhizic acid have been investigated for potentially beneficial antioxidant, anti-inflammatory, and hepatoprotective effects [40]. Conversely, consistent or excessive licorice ingestion has been associated with hypertension and hypokalemia [42]. Recent clinical evidence further demonstrates that licorice exposure may affect renin, aldosterone, and blood pressure, even at comparatively low daily doses [73], while reported cases continue to illustrate the risk of licorice-induced pseudohyperaldosteronism [74]. Similarly, the chemical composition of *G. lucidum* products may vary according to species, geographical origin, cultivation conditions, and processing, thereby affecting product consistency [49]. Herbal and fungal products may also be affected by adulteration, undeclared substitution, or species misidentification [53,55,56]. Pharmacopoeial marker-compound thresholds and taxonomic authentication are therefore important, but they may not fully capture complex chemical fingerprints, interactions among constituents, or biological potency. Controlled plant-cell culture and fungal fermentation may improve supply stability and control over production conditions [57,60,61]; however, improvements in production yield or reductions in manufacturing cost do not by themselves demonstrate equivalent biological activity. Future product development should combine botanical or fungal authentication, multi-component chemical fingerprinting, contaminant testing, stability assessment, and mechanism-relevant potency assays [52]. Broader reviews of medicine–food homology substances similarly emphasize the importance of constituent characterization, standardization, and safety evaluation for reproducible food and health applications [3].

This review has several limitations. The evidence base was small, heterogeneous, and predominantly preclinical, with only four randomized clinical studies; these features precluded quantitative synthesis and limited direct comparison across studies. The review protocol was not prospectively registered. In addition, the restriction to English-language reports may have introduced language bias. Accordingly, the findings should be interpreted as a structured synthesis of mechanistic and translational evidence rather than as definitive evidence of anti-aging efficacy in humans.

Future research should directly address the gaps identified in the present evidence base. Preclinical studies should use well-characterized plant materials, prespecified doses, appropriate aging models, adequate randomization and blinding, and outcomes that distinguish disease-specific protection from modification of fundamental aging processes. Delivery-system studies should determine whether improvements in stability or exposure produce corresponding changes in molecular pathways and clinically relevant outcomes [65,75]. They should also assess food-grade carrier safety, gastrointestinal transformation, tissue distribution, long-term accumulation, and compliance with particle-specific regulatory requirements [68]. Human studies should be prospectively registered, adequately powered, multicenter, and sufficiently long to evaluate durability and safety. Trial outcomes should combine validated biomarkers with physical function, cognitive function, frailty, quality of life, and the incidence or progression of age-related disease [69]. Selection of biomarker panels should consider complementary physiological, inflammatory, functional, and epigenetic dimensions [70]. Dose–response relationships, sex-specific effects, baseline metabolic status, product composition, and adherence should be prespecified rather than explored only after data collection. Multi-omics and microbiome analyses may help identify responders and clarify mechanisms, but these approaches should be independently validated and should not replace clinically meaningful endpoints. Collectively, these priorities would provide a more reliable basis for determining whether medicinal and edible plant-derived compounds can contribute to healthy aging beyond short-term changes in molecular or metabolic biomarkers.

## 5. Conclusions

In summary, the 12 eligible primary studies suggest that selected bioactive compounds from medicinal and edible sources may modulate aging-related processes involving mitochondrial function, antioxidant defense, metabolic regulation, cytoskeletal stability, gut–brain signaling, and extracellular-matrix maintenance. The three delivery-system/formulation studies indicate that nanoliposomes and poly(lactic-co-glycolic acid) nanoparticles may improve selected stability, penetration, cellular uptake, and pharmacokinetic outcomes; however, these formulation advantages have not been shown to translate into durable anti-aging benefits. The evidence base was small, heterogeneous, and predominantly preclinical. Human evidence was limited to four randomized clinical studies, several with small samples or short follow-up, and certainty ranged from moderate to very low. Accordingly, the findings should be interpreted as mechanistically supportive and translationally promising rather than as definitive evidence of anti-aging efficacy in humans. Future research should prioritize standardized plant materials and aging-related outcomes, mechanism-linked evaluation of food-grade delivery systems, adequately powered long-term registered trials, and clinically meaningful measures of physical function, cognition, frailty, quality of life, and age-related disease.

## Figures and Tables

**Table 1:** Summary of characteristics of included human clinical trials.

Intervention/ Active Ingredient	Study Design	Population/ Sample	Duration	Primary Outcomes	Key Findings	Ref.
TA-65 (*Astragalus* *membranaceus* extract; cycloastragenol derivative)	Randomized double-blind placebo- controlled trial.	117 healthy CMV- positive subjects (53–87 years).	12 months	Leukocyte telomere length.	250 U/day: telomere length (+530 ± 180 bp); placebo: telomere length (−290 ± 100 bp).	[26]
Resveratrol	Randomized double-blind placebo- controlled crossover trial.	11 healthy obese men (52 ± 5 years).	30 days	Resting metabolic rate; blood pressure; blood glucose; blood lipids.	resting metabolic rate, systolic blood pressure, blood glucose, triglycerides and liver lipids.	[27]
Resveratrol	Prospective open-label randomized controlled trial.	62 patients with type 2 diabetes mellitus; 32 control and 30 intervention.	3 months	HbA1c; fasting blood glucose; blood pressure; lipid profile; renal/protein markers.	250 mg/day plus oral hypoglycemic agents: HbA1c, systolic blood pressure, total cholesterol and total protein; fasting blood glucose and LDL-C showed downward trends.	[28]
trans-Resveratrol	Randomized double-blind placebo- controlled trial.	19 male patients with type 2 diabetes mellitus; 10 resveratrol and 9 placebo.	4 weeks	Insulin sensitivity; urinary ortho-tyrosine; incretins; platelet pAkt/Akt ratio.	2 × 5 mg/day: HOMA-IR and urinary ortho-tyrosine; platelet pAkt/Akt ratio; no significant β-cell-related effect.	[29]

Abbreviations: CMV, cytomegalovirus; HbA1c, glycated hemoglobin; HOMA-IR, Homeostatic Model Assessment for Insulin Resistance; LDL-C, low-density lipoprotein cholesterol; pAkt, Phosphorylated protein kinase B; Akt, Protein kinase B.

**Table 2:** Summary of characteristics of included animal studies.

Plant/ Component	Active Ingredient	Animal Model	Sample Size	Intervention	Primary Outcomes	Key Findings	Ref.
Resveratrol	Resveratrol	C57BL/6J mice with high-fat diet-induced obesity.	8 per group.	400 mg/kg/day dietary supplementation for 15 weeks.	Running endurance; muscle mitochondrial function.	Running time 57%; muscle mitochondrial number; PGC-1α acetylation.	[25]
*Astragalus* *membranaceus*	Astragaloside IV	Male Sprague-Dawley rats; streptozotocin- induced diabetic nephropathy.	10 normal, 14 DN model, 14 AS-IV group.	40 mg/kg/day intragastric administration for 12 weeks.	Blood glucose; urinary microalbumin; renal histopathology; podocyte adhesion- related proteins.	blood glucose and urinary microalbumin; improved renal pathology; regulated β1- integrin, ILK and α-actinin-4.	[30]
*Ganoderma* *lucidum*	*Ganoderma* *lucidum* polysaccharide	D-galactose- induced aging mice.	*n* = 10/group for behavioral testing; biochemical assays *n* = 3/group.	GLP intervention in D- galactose- induced aging mice.	Morris water maze; SOD, GSH-Px, MDA; Glu, ACh, GABA; 16S rRNA sequencing.	Improved learning/memory; antioxidant capacity; lipid peroxidation/oxidative stress; modulated intestinal flora, especially *Lactobacillus*.	[31]
Betulinic acid	Betulinic acid (BetA)	Wild-type *Drosophila* *melanogaster* (wCS10); Sir2-deficient and FoxO-null mutant flies.	Lifespan assay: *n* = 300/sex/group.	10–100 μM dietary BetA supplementation throughout adulthood; 50 μM for subsequent experiments.	Mean and maximum lifespan; mortality; physiological function; stress resistance; Sir2/FoxO dependence.	50 μM BetA increased mean lifespan by 13% in males and 6% in females; enhanced oxidative-stress resistance; increased *sir2* expression; no lifespan extension in Sir2- deficient or FoxO-null mutants.	[32]

Abbreviations: ACh, acetylcholine; AS-IV, astragaloside IV; BetA, betulinic acid; DN, diabetic nephropathy; FoxO, forkhead box O; GABA, γ-aminobutyric acid; GLP, *Ganoderma lucidum* polysaccharide; GSH-Px, glutathione peroxidase; ILK, integrin-linked kinase; MDA, malondialdehyde; Sir2, silent information regulator 2; SOD, superoxide dismutase; Glu, Glutamate; 16SrRNA, 16S ribosomal RNA.

**Table 3:** Summary of molecular mechanisms of major active ingredients.

Compound Class	Representative Component/Source	Targeted Pathway	Key Molecular Events	Biological Relevance	Ref.
Stilbenoids	Resveratrol/grape	SIRT1/PGC-1α	Activates SIRT1 deacetylase activity; reduces PGC-1α acetylation.	mitochondrial biogenesis; improved insulin sensitivity.	[25]
Stilbenoids	Resveratrol/resVida trans-resveratrol supplement	AMPK/SIRT1/ PGC-1α	Activates AMPK; SIRT1 and PGC-1α; citrate synthase activity and mitochondrial respiration.	Improved metabolic profile; glucose, triglycerides, liver lipids and HOMA index.	[27]
Saponins	Astragaloside IV/*Astragalus* *membranaceus*	β1-integrin/ILK signaling	Regulates β1-integrin and integrin-linked kinase expression in renal tissue.	Improved podocyte adhesion; urine albumin; delayed diabetic nephropathy progression.	[30]
Saponins	Astragaloside IV/*Astragalus* *membranaceus*.	α-actinin-4/ podocyte cytoskeletal regulation.	Modulates α-actinin-4 expression and improves renal pathological changes.	Protected glomerular structure; blood glucose and urinary microalbumin.	[30]
Polysaccharides	*Ganoderma* *lucidum* polysaccharide/*Ganoderma lucidum*.	Antioxidant defense/ gut–brain axis.	SOD and GSH-Px; Glu and ACh; MDA and GABA; modulated *Lactobacillus*.	Improved learning/memory; oxidative stress; regulated intestinal flora in D-galactose-induced aging mice.	[31]
Flavonoids	EGCG/green tea	MAPK-mediated MMP/collagen degradation pathway.	Inhibits UVB-induced ASK-1 activation and JNK, p38 MAPK and ERK1/2 phosphorylation; suppresses MMP-1, MMP-8 and MMP-13.	Reduced collagen destruction; protected dermal fibroblasts from UVB-induced photoaging damage.	[33]

Abbreviations: ACh, acetylcholine; AMPK, AMP-activated protein kinase; ASK-1, apoptosis signal-regulating kinase 1; EGCG, epigallocatechin-3-gallate; ERK, extracellular signal-regulated kinase; GABA, γ-aminobutyric acid; GSH-Px, glutathione peroxidase; ILK, integrin-linked kinase; JNK, c-Jun N-terminal kinase; MAPK, mitogen-activated protein kinase; MDA, malondialdehyde; MMP, matrix metalloproteinase; PGC-1α, peroxisome proliferator-activated receptor gamma coactivator 1-alpha; SIRT1, sirtuin 1; SOD, superoxide dismutase; UVB, ultraviolet B; Glu, Glutamate.

**Table 4:** Summary of characteristics of delivery-system studies.

Delivery System	Encapsulated Component	Formulation Parameters	Study Context	Main Effects	Ref.
Nanoliposomes	Coffee berry extract	Size: 117.33 ± 2.91 nm; zeta potential: −56.13 ± 1.87 mV; EE: 71.26 ± 3.12%.	In vitro + in vivo skin evaluation.	Improved phenolic stability and sustained release; SOD and anti-collagenase activity; improved skin elasticity and brightness.	[7]
Nanoliposomes	Resveratrol	Size: 55.6 ± 3.2 nm; zeta potential: −20.3 ± 0.8 mV; EE: 90.9 ± 1.6%.	In vitro + 3D skin model + in vivo human skin study.	Improved transdermal penetration/skin retention and cellular uptake; increased antioxidant capacity and collagen synthesis; decreased MMP secretion; anti-wrinkle/brightening effects.	[8]
PLGA nanoparticles	Curcumin	Size: ~200 nm; zeta potential: not reported; EE: 91.96%.	In vitro + in vivo animal pharmacokinetic study.	Improved water solubility (~640-fold); sustained intestinal release; oral relative bioavailability (5.6-fold); prolonged plasma half-life.	[34]

Abbreviations: EE, encapsulation efficiency; MMP, matrix metalloproteinase; PLGA, poly(lactic-co-glycolic acid); SOD, superoxide dismutase.

**Table 5:** Risk of bias assessment of randomized clinical trials using the Cochrane RoB 2 tool.

Study	D1	D2	D3	D4	D5	Overall
Salvador et al. [26]	SC	L	H	L	SC	H
Timmers et al. [27]	SC	L	L	L	H	H
Bhatt et al. [28]	H	SC	L	L	SC	H
Brasnyó et al. [29]	SC	L	L	L	H	H

Notes: D1, randomization process; D2, deviations from intended interventions; D3, missing outcome data; D4, outcome measurement; D5, selection of the reported result. L, low risk; SC, some concerns; H, high risk.

**Table 6:** GRADE assessment of the evidence for medicinal and edible plant-derived interventions on anti-aging-related clinical outcomes.

Study	Outcome	Design	Risk of Bias	Inconsistency	Indirectness	Imprecision	Other Considerations	Certainty	Importance
Salvador et al. 2016	Leukocyte telomere length	Randomized trial	Serious: missing outcome data; concerns in randomization/ selective reporting.	Not serious	Not serious: direct population, intervention, comparator and outcome.	Serious: 117 recruited, 97 completed; single trial.	Strong association	Moderate	Critical
Timmers et al. 2011	Resting metabolic rate	Randomized trial	Serious: selective reporting concerns and some concerns in randomization.	Not serious	Not serious: direct metabolic adaptation measure.	Very serious: *n* = 11; unstable estimates.	None	Very low	Important
Timmers et al. 2011	Systolic blood pressure	Randomized trial	Serious: selective reporting concerns and some concerns in randomization.	Not serious	Not serious: direct cardiometabolic outcome.	Very serious: *n* = 11; limited precision.	None	Very low	Important
Timmers et al. 2011	Blood glucose	Randomized trial	Serious: selective reporting concerns and some concerns in randomization.	Not serious	Not serious: direct metabolic/glycemic outcome.	Very serious: *n* = 11; high uncertainty.	None	Very low	Critical
Timmers et al. 2011	Triglycerides	Randomized trial	Serious: selective reporting concerns and some concerns in randomization.	Not serious	Not serious: direct metabolic outcome.	Very serious: *n* = 11; serious uncertainty.	None	Very low	Important
Timmers et al. 2011	Liver lipids	Randomized trial	Serious: selective reporting concerns and some concerns in randomization.	Not serious	Serious: surrogate metabolic marker.	Very serious: *n* = 11; imprecise estimate.	None	Very low	Important
Bhatt et al. 2012	HbA1c	Randomized trial	Serious: open-label design; concerns in randomization/ intervention deviations.	Not serious	Not serious: clinically relevant glycemic outcome.	Serious: modest sample; 57/62 analyzed at follow-up.	None	Low	Critical
Bhatt et al. 2012	Fasting blood glucose	Randomized trial	Serious: open-label design; concerns in randomization/ intervention deviations.	Not serious	Not serious: direct metabolic/glycemic outcome.	Serious: modest sample and incomplete follow-up	None	Low	Critical
Bhatt et al. 2012	Systolic blood pressure	Randomized trial	Serious: open-label design; concerns in randomization/ intervention deviations.	Not serious	Not serious: direct cardiometabolic outcome.	Serious: modest sample and limited follow-up.	None	Low	Important
Bhatt et al. 2012	Lipid profile	Randomized trial	Serious: open-label design; concerns in randomization/ intervention deviations.	Not serious	Not serious: relevant to cardiometabolic risk.	Serious: modest sample; some estimates not robust.	None	Low	Important
Brasnyó et al. 2011	HOMA-IR	Randomized trial	Serious: selective reporting concerns and some concerns in randomization	Not serious	Not serious: direct insulin-resistance marker	Very serious: *n* = 19; substantial uncertainty	None	Very low	Critical
Brasnyó et al. 2011	Urinary ortho- tyrosine	Randomized trial	Serious: selective reporting concerns and some concerns in randomization	Not serious	Not serious: oxidative stress marker relevant to anti- aging mechanism	Very serious: *n* = 19; limited certainty	None	Very low	Important
Brasnyó et al. 2011	Platelet pAkt/Akt ratio	Randomized trial	Serious: selective reporting concerns and some concerns in randomization	Not serious	Serious: mechanistic surrogate marker	Very serious: *n* = 19; highly uncertain estimate	None	Very low	Important

Abbreviations: GRADE, Grading of Recommendations Assessment, Development and Evaluation; HbA1c, glycated hemoglobin; HOMA-IR, Homeostatic Model Assessment for Insulin Resistance; pAkt, Phosphorylated protein kinase B; Akt, Protein kinase B.

**Table 7:** Risk of bias assessment of the included animal studies using the SYRCLE risk-of-bias tool.

Study	SG	BC	AC	RH	BI/C	ROA	BOA	IOD	SOR	OSB
Lagouge et al. [25]	U	L	U	U	U	U	U	L	U	H
Lu et al. [30]	U	L	U	U	U	L	U	H	U	L
Chen et al. [31]	U	L	U	U	U	H	U	H	U	H
Lee et al. [25]	U	L	U	U	U	L	U	L	L	H

Notes: SG, sequence generation; BC, baseline characteristics; AC, allocation concealment; RH, random housing; BI/C, blinding of investigators/caregivers; ROA, random outcome assessment; BOA, blinding of outcome assessors; IOD, incomplete outcome data; SOR, selective outcome reporting; OSB, other sources of bias. L, low risk; U, unclear risk; H, high risk.

**Table 8:** Risk of bias assessment of the included delivery-system/formulation studies using a review-specific domain-based checklist informed by the NTP/OHAT systematic-review framework.

Study	Study Type	D/E	GA	EC	OD	EFC	AB	OR	OB
Saewan et al. [7]	In vitro + in vivo	L	L	L	L	L	U	L	L
Zhang et al. [8]	In vitro + 3D skin model + in vivo human skin study	L	L	L	L	L	U	L	H
Xie et al. [34]	In vitro + in vivo animal pharmacokinetic study	L	L	L	L	L	U	L	L

Notes: L, low risk; U, unclear risk; H, high risk. Detailed operational criteria for each domain and judgment category are provided in Supplementary Table S3. Abbreviations: AB, assessor blinding; D/E, dose or exposure administration; EC, comparability of experimental conditions; EFC, exposure or formulation characterization; GA, group allocation; OB, other potential threats to internal validity; OD, completeness of outcome data; OR, completeness of outcome reporting.

## Data Availability

No new data were created or analyzed in this study. Data sharing is not applicable to this article.

## Supplementary Materials

The following supporting information can be downloaded at: https://www.mdpi.com/article/10.3390/foods15152646/s1, Table S1: PRISMA 2020 checklist for this systematic review. Table S2. Study-level characteristics, principal-objective classification, and methodological appraisal of the 12 included primary studies. Table S3. Operational criteria for the review-specific domain-based checklist used to assess risk of bias in delivery-system/formulation studies. Refs. [7,8,15,16,17,25,26,27,28,29,30,31,32,33,34] are cited in the Supplementary Materials.

## Abbreviations

The following abbreviations are used in this manuscript:

16S rRNA	16S ribosomal RNA
ACh	Acetylcholine
Akt	Protein kinase B
AMPK	Adenosine monophosphate-activated protein kinase
ARE	Antioxidant response element
AS-IV	Astragaloside IV
ASK-1	Apoptosis signal-regulating kinase 1
BetA	Betulinic acid
CMV	Cytomegalovirus
DN	Diabetic nephropathy
EGCG	Epigallocatechin-3-gallate
ERK	Extracellular signal-regulated kinase
FoxO	Forkhead box O
GABA	Gamma-aminobutyric acid
GLP	*Ganoderma lucidum* polysaccharide
Glu	Glutamate
GRADE	Grading of Recommendations Assessment, Development and Evaluation
GSH-Px	Glutathione peroxidase
HbA1c	Glycated hemoglobin
HOMA-IR	Homeostatic Model Assessment for Insulin Resistance
ILK	Integrin-linked kinase
JNK	c-Jun N-terminal kinase
LDL-C	Low-density lipoprotein cholesterol
MAPK	Mitogen-activated protein kinase
MDA	Malondialdehyde
MMP	Matrix metalloproteinase
NF-κB	Nuclear factor kappa B
Nrf2	Nuclear factor erythroid 2-related factor 2
NTP/OHAT	National Toxicology Program/Office of Health Assessment and Translation
pAkt	Phosphorylated protein kinase B
PGC-1α	Peroxisome proliferator-activated receptor gamma coactivator 1-alpha
PLGA	Poly(lactic-co-glycolic acid)
PRISMA	Preferred Reporting Items for Systematic Reviews and Meta-Analyses
RoB 2	Cochrane Risk of Bias 2 tool
ROBINS-I	Risk Of Bias In Non-randomized Studies-of Interventions
Sir2 SIRT1	Silent information regulator 2 Sirtuin 1
SOD	Superoxide dismutase
SYRCLE	Systematic Review Centre for Laboratory Animal Experimentation
UVB	Ultraviolet B

## Appendix A

**Table A1:** Database search strategy for anti-aging active ingredients of medicinal and edible plants.

Database	Search Query Components	Applied Filters	Syntax/Modifiers
PubMed	(Medicinal/edible plants OR phytochemicals) AND (aging OR anti-aging OR longevity) AND ( oxidative stress OR inflammation OR telomere OR sirtuin OR AMPK OR autophagy OR clinical trial)	English; 1993–2025	((“Plants, Medicinal”[Mesh] OR “Plants, Edible”[Mesh] OR medicinal plant[tiab] OR edible plant[tiab] OR phytochemical[tiab] OR herb[tiab]) AND (“Aging”[Mesh] OR “Longevity”[Mesh] OR anti-aging[tiab] OR senescence[tiab]) AND (“Oxidative Stress”[Mesh] OR “Inflammation”[Mesh] OR telomere[tiab] OR sirtuin[tiab] OR AMPK[tiab] OR autophagy[tiab] OR clinical trial[tiab]))
Scopus	(medicinal/edible plants OR phytochemicals) AND (aging OR anti-aging OR molecular mechanisms OR delivery systems OR clinical trials)	English; 1993–2025	TITLE-ABS-KEY((medicinal plant* OR edible plant* OR phytochemical* OR herb*) AND (aging OR anti-aging OR longevity OR senescence) AND (oxidative stress OR inflammation OR telomere OR sirtuin OR AMPK OR autophagy OR nano-delivery OR clinical trial))
Embase	(medicinal/edible plants OR phytochemicals) AND (aging OR anti-aging OR longevity) AND ( oxidative stress OR inflammation OR telomere OR sirtuin OR AMPK OR autophagy)	English; 1993–2025	(‘medicinal plant’/exp OR ‘edible plant’/exp OR ‘ phytochemistry’/exp OR medicinal plant:ti,ab OR edible plant:ti,ab OR phytochemical:ti,ab) AND (‘aging’/exp OR ‘longevity’/exp OR anti-aging:ti,ab OR senescence:ti,ab) AND (‘oxidative stress’/exp OR ‘inflammation’/exp OR telomere:ti,ab OR sirtuin:ti,ab OR AMPK:ti,ab OR autophagy:ti,ab)
Web of Science	(medicinal/edible plants OR phytochemicals) AND (anti-aging mechanisms OR molecular pathways OR clinical evidence)	English; 1993–2025	TS = ((medicinal plant* OR edible plant* OR phytochemical* OR herb*) AND (aging OR anti-aging OR longevity OR senescence) AND (oxidative stress OR inflammation OR telomere OR sirtuin OR AMPK OR autophagy OR clinical trial))

Note: The asterisk (*) denotes a truncation wildcard used to retrieve multiple word variants sharing the same root. Abbreviations: AMPK, AMP-activated protein kinase.
