# Supplementary material for: Bioactive Compounds from Medicinal and Edible Plants for Anti-Aging: A Systematic Review of Molecular Mechanisms, Delivery Systems, and Clinical Evidence
Source: Foods. 2026 Jul 28;15(15):2646. doi: 10.3390/foods15152646 (PMC13466083; doi:10.3390/foods15152646)
Supplement: Supplementary file 1 [file foods-15-02646-s001.zip › Supplementary Table S2. Study-level characteristics, principal-objective classification, and methodological appraisal of the 12 included primary studies..pdf]

Table S2. Study-level characteristics, principal-objective classification, and methodological appraisal of the 12 included primary studies.

Study	Bioactive material/intervention	Study design or model	Intervention/exposure	Principal outcomes and findings	Principal-objective classification	Methodological appraisal
Salvador et al., 2016	TA-65, an *Astragalus membranaceus*-derived telomerase activator	Randomized, double-blind, placebo-controlled study; 117 healthy cytomegalovirus-positive adults aged 53–87 years	TA-65, including 250 U/day, versus placebo for 12 months	Leukocyte telomere length increased in the 250 U/day group, whereas a decrease was reported in the placebo group	Evaluation of a telomere-related outcome in human participants; randomized clinical study	Cochrane Risk of Bias 2; clinical-outcome certainty also assessed using GRADE
Timmers et al., 2011	Resveratrol	Randomized, double-blind, placebo-controlled crossover study; 11 men with obesity	Resveratrol versus placebo for 30 days	Reported reductions in resting metabolic rate, systolic blood pressure, blood glucose, triglycerides, and liver lipids, together with changes in mitochondrial and metabolic measures	Evaluation of metabolic effects in human participants; randomized clinical study	Cochrane Risk of Bias 2; clinical-outcome certainty also assessed using GRADE

Study	Bioactive material/intervention	Study design or model	Intervention/exposure	Principal outcomes and findings	Principal-objective classification	Methodological appraisal
Bhatt et al., 2012	Resveratrol	Prospective, open-label, randomized controlled study; 62 patients with type 2 diabetes mellitus	Resveratrol, 250 mg/day, plus oral hypoglycemic treatment versus standard treatment for 3 months	Improvements were reported in glycated hemoglobin, systolic blood pressure, total cholesterol, and total protein; fasting glucose and low-density lipoprotein cholesterol showed downward trends	Evaluation of glycemic and cardiometabolic effects in human participants; randomized clinical study	Cochrane Risk of Bias 2; clinical-outcome certainty also assessed using GRADE
Brasnyó et al., 2011	trans-Resveratrol	Randomized, double-blind, placebo-controlled study; 19 men with type 2 diabetes mellitus	trans-Resveratrol, 2 × 5 mg/day, versus placebo for 4 weeks	Reduced homeostatic model assessment of insulin resistance and urinary ortho-tyrosine; increased platelet phosphorylated Akt-to-total Akt ratio; no significant β-cell-related effect	Evaluation of insulin sensitivity and oxidative-stress-related outcomes in human participants; randomized clinical study	Cochrane Risk of Bias 2; clinical-outcome certainty also assessed using GRADE

Study	Bioactive material/intervention	Study design or model	Intervention/exposure	Principal outcomes and findings	Principal-objective classification	Methodological appraisal
Lagouge et al., 2006	Resveratrol	C57BL/6J mice with high-fat-diet-induced obesity; eight animals per group	Dietary resveratrol, 400 mg/kg/day, for 15 weeks	Increased running endurance and skeletal-muscle mitochondrial content; associated with sirtuin 1/peroxisome proliferator-activated receptor gamma coactivator 1-alpha regulation	Evaluation of metabolic and mitochondrial effects in an animal model; animal study	Systematic Review Centre for Laboratory Animal Experimentation risk-of-bias tool
Lu et al., 2015	Astragaloside IV from *Astragalus membranaceus*	Male Sprague–Dawley rats with streptozotocin-induced diabetic nephropathy; normal, disease-model, and astragaloside IV groups	Astragaloside IV, 40 mg/kg/day by intragastric administration, for 12 weeks	Reduced blood glucose and urinary microalbumin, improved renal pathology, and regulated β1-integrin, integrin-linked kinase, and α-actinin-4	Evaluation of renal protection and podocyte-related mechanisms in an animal model; animal study	Systematic Review Centre for Laboratory Animal Experimentation risk-of-bias tool
Chen et al., 2025	*Ganoderma lucidum* polysaccharide	D-galactose-induced aging mice; 10 animals per group for behavioral testing and three per group for biochemical assays	*G. lucidum* polysaccharide intervention in D-galactose-treated mice	Improved learning and memory, antioxidant status, and lipid-peroxidation-related indicators; altered intestinal microbiota, particularly *Lactobacillus* abundance	Evaluation of behavioral, antioxidant, and gut–brain-axis effects in an animal model; animal study	Systematic Review Centre for Laboratory Animal Experimentation risk-of-bias tool

Study	Bioactive material/intervention	Study design or model	Intervention/exposure	Principal outcomes and findings	Principal-objective classification	Methodological appraisal
Lee and Min, 2024	Betulinic acid	Wild-type *Drosophila melanogaster* and Sir2-deficient and FoxO-null mutants; lifespan assay, 300 flies per sex per group	Dietary betulinic acid at 10–100 μM throughout adulthood; 50 μM used in subsequent experiments	At 50 μM, mean lifespan increased by 13% in males and 6% in females; oxidative-stress resistance and *sir2* expression increased; lifespan was not extended in Sir2-deficient or FoxO-null mutants	Evaluation of lifespan and Sir2/FoxO dependence in an animal model; animal study	Systematic Review Centre for Laboratory Animal Experimentation risk-of-bias tool
Bae et al., 2008	Epigallocatechin-3-gallate	Ultraviolet B-exposed human dermal fibroblasts	Epigallocatechin-3-gallate treatment in an ultraviolet B-induced photoaging-related cell model	Reduced collagen destruction and collagenase activation by suppressing mitogen-activated protein kinase signaling and matrix metalloproteinase-1, -8, and -13	Direct investigation of a molecular mechanism in a cell model; standalone in vitro mechanistic study	Narrative methodological appraisal; no formal study-design-specific tool was prespecified

Study	Bioactive material/intervention	Study design or model	Intervention/exposure	Principal outcomes and findings	Principal-objective classification	Methodological appraisal
Saewan et al., 2023	Coffee berry extract nanoliposomes	In vitro formulation assays and in vivo skin evaluation	Nanoliposomal coffee berry extract compared with non-encapsulated or control preparations	Improved phenolic stability and sustained release, antioxidant and anti-collagenase activity, skin elasticity, and skin brightness	Primary objective was formulation development and performance evaluation; delivery-system/formulation study	Review-specific domain-based checklist informed by the National Toxicology Program/Office of Health Assessment and Translation framework
Zhang et al., 2023	Resveratrol nanoliposomes	In vitro assays, three-dimensional skin model, and in vivo human skin evaluation	Nanoliposomal resveratrol compared with relevant non-encapsulated or control preparations	Improved transdermal penetration, skin retention, and cellular uptake; increased antioxidant capacity and collagen synthesis; reduced matrix metalloproteinase secretion; reported anti-wrinkle and brightening effects	Primary objective was transdermal formulation development and performance evaluation; delivery-system/formulation study	Review-specific domain-based checklist informed by the National Toxicology Program/Office of Health Assessment and Translation framework

Study	Bioactive material/intervention	Study design or model	Intervention/exposure	Principal outcomes and findings	Principal-objective classification	Methodological appraisal
Xie et al., 2011	Curcumin-loaded poly(lactic-co-glycolic acid) nanoparticles	In vitro formulation studies and in vivo rat pharmacokinetic study	Nanoparticle-encapsulated curcumin compared with native curcumin	Approximately 640-fold improvement in water solubility, sustained intestinal release, 5.6-fold increase in relative oral bioavailability, and prolonged plasma half-life	Primary objective was formulation and pharmacokinetic enhancement; delivery-system/formulation study	Review-specific domain-based checklist informed by the National Toxicology Program/Office of Health Assessment and Translation framework

Notes: Each study was assigned to one mutually exclusive category according to its principal research objective and was counted only once. Delivery-system/formulation studies containing in vitro assays, three-dimensional skin models, human skin evaluations, or animal pharmacokinetic components were classified according to their formulation-focused principal objective rather than being counted again as separate in vitro, clinical, or animal studies. Table 3 in the main manuscript represents a cross-study mechanistic synthesis and does not constitute an additional category of included studies.

Abbreviations: Akt, protein kinase B; FoxO, forkhead box O; GRADE, Grading of Recommendations Assessment, Development and Evaluation; Sir2, silent information regulator 2; TA-65, an *Astragalus membranaceus*-derived telomerase activator.
