# Supplementary material for: Bioactive Compounds from Medicinal and Edible Plants for Anti-Aging: A Systematic Review of Molecular Mechanisms, Delivery Systems, and Clinical Evidence
Source: Foods. 2026 Jul 28;15(15):2646. doi: 10.3390/foods15152646 (PMC13466083; doi:10.3390/foods15152646)
Supplement: Supplementary file 1 [file foods-15-02646-s001.zip › Supplementary Table S3. Operational criteria for the review-specific domain-based checklist used to assess risk of bias in delivery-systemformulation studies..pdf]

Table S3. Operational criteria for the review-specific domain-based checklist used to assess risk of bias in delivery-system/formulation studies.

Domain	Low risk	Unclear risk	High risk
Dose or exposure administration	Dose, concentration, route, duration, frequency, and vehicle or control conditions were adequately described and consistently administered.	One or more relevant administration details were insufficiently reported.	Administration differed systematically between groups, was inadequately controlled, or was likely to cause exposure misclassification.
Group allocation	Experimental units or samples were assigned using a random or otherwise clearly unbiased procedure, or the allocation process was unlikely to influence the findings.	The allocation procedure was not adequately described.	Allocation was demonstrably non-random or produced systematically non-comparable groups.
Comparability of experimental conditions	Groups were maintained under comparable conditions apart from the intervention or formulation being evaluated.	Reporting was insufficient to determine whether experimental conditions were comparable.	Important differences in culture, handling, vehicle, housing, treatment, or follow-up conditions were present between groups.
Completeness of outcome data	Outcome data were complete, or missing observations and exclusions were limited, balanced, and adequately explained.	Missing data or exclusions were insufficiently described.	Substantial or differential missing data or unexplained exclusions were likely to affect the findings.
Exposure or formulation characterization	The active compound, formulation, concentration, particle properties, encapsulation, stability, or exposure level was characterized using appropriate methods, as relevant to the study design.	Characterization was incomplete or insufficiently described.	Exposure or formulation characterization was inadequate or unreliable and was likely to compromise interpretation.

Domain	Low risk	Unclear risk	High risk
Assessor blinding	Assessors were blinded, or outcomes were measured using sufficiently objective or automated methods unlikely to be influenced by assessor knowledge.	Blinding was not reported and the influence of assessor knowledge could not be determined.	Assessors were not blinded for subjective outcomes, and knowledge of group allocation was likely to influence assessment.
Completeness of outcome reporting	All outcomes described in the Methods were reported, or there was no indication of selective omission.	It was unclear whether all measured outcomes were reported.	One or more prespecified or clearly measured outcomes were selectively omitted or incompletely reported.
Other potential threats to internal validity	Statistical analyses, controls, unit-of-analysis decisions, and other methodological features were appropriate, with no additional major concern identified.	Information was insufficient to exclude other important sources of bias.	A major additional concern—such as inappropriate statistics, lack of an essential control, pseudoreplication, or a substantial conflict affecting study conduct—was identified.
